# The prevalence and epidemiology of genetic renal disease amongst adults with chronic kidney disease in Australia

**DOI:** 10.1186/1750-1172-9-98

**Published:** 2014-06-30

**Authors:** Andrew Mallett, Chirag Patel, Anne Salisbury, Zaimin Wang, Helen Healy, Wendy Hoy

**Affiliations:** 1 Department of Renal Medicine, Royal Brisbane and Women’s Hospital, Brisbane, Australia; 2 Centre for Kidney Disease Research, School of Medicine, University of Queensland, Brisbane, Australia; 3 CKD.QLD and Centre for Chronic Disease, School of Medicine, University of Queensland, Brisbane, Australia; 4 Genetic Health Queensland, Royal Brisbane and Women’s Hospital, Brisbane, Australia

**Keywords:** Genetic renal disease, Chronic kidney disease, Nephrology, Nephrogenetics, Epidemiology

## Abstract

**Background:**

There are an established and growing number of Mendelian genetic causes for chronic kidney disease (CKD) in adults, though estimates of prevalence have been speculative. The CKD Queensland (CKD.QLD) registry enables partial clarification of this through the study of adults with CKD receiving nephrology care throughout Queensland, Australia.

**Methods:**

Data from the first 2,935 patients consented to the CKD.QLD registry across five sites was analysed, with a comparison between those with and without Genetic Renal Disease (GRD). Prevalence of GRD amongst those with diagnosed CKD, the general population, and commencing renal replacement therapy (RRT) was calculated using the CKD.QLD registry, national census data and extracted Australian and New Zealand Dialysis and Transplantation (ANZDATA) registry report data respectively.

**Results:**

Patients with GRD constituted 9.8% of this Australian adult CKD cohort (287/2935). This was lower than in local incident RRT cohorts (2006–2011: 9.8% vs 11.3%, x^2^ = 0.014). Cases of adult CKD GRD were more likely to be female (54.0% vs 45.6%; x^2^ = 0.007), younger (mean 52.6 yrs vs 69.3 yrs, p < 0.001), have a higher eGFR (mean 49.7 ml/min/1.73 m2 vs 40.4 ml/min/1.73 m2, p < 0.001), and have earlier stage renal disease (CKD Stage 1: 15.7% vs 5.1%, x^2^ < 0.0005) than those without GRD.

**Conclusions:**

The proportion of GRD amongst an Australian adult CKD population in specialty renal practice is similar to past estimations. GRD is a significant cause for CKD and for RRT commencement, presenting opportunities for ongoing longitudinal study, directed therapeutics and clinical service redesign.

## Background

Chronic Kidney Disease (CKD) is common, being present in approximately 1 in 7 Australians aged 25 years or older [1]. Whilst etiologically heterogeneous, up to 20% of cases of CKD are thought to be due to genetic forms of renal disease [2-4]. This approximates to a population prevalence with Genetic Renal Disease (GRD) of 32,000/million aged ≥25 years, which, in absolute numbers represents 422,842 Australians or 3.2% of the Australian population ≥25 years in 2011 (http://www.abs.gov.au). This population shares the higher morbidity, mortality and health care requirements [5] common to all patients with CKD. It is unclear however whether CKD due to various forms of GRD carries the same risks as that associated with other CKD aetiologies. This requires longitudinal studies and further clarification. Admissions for those progressing to end stage kidney disease (ESKD) requiring renal replacement therapy (RRT), alone are presently the most common cause for hospital admission in Australia [6,7]. Of the 2,453 patients who commenced RRT in Australia during 2011, GRD was the cause for ESKD in 13% [8].

The prevalence of GRD in the Australian paediatric population is 70.6/million aged <20 years [9], though this may be an underestimate. The GRD spectrum is vast, encompassing many different diseases and disease groupings. Causative genetic and pathobiological understanding of some is advanced, as in ADPKD [10-13] and Alport Syndrome [14-18]. For many other forms of GRD such as ciliopathies [19,20] and congenital anomalies of the kidney and urinary tract (CAKUT) [21-26] this is much less so but continues to expand every year. Emerging research tools such as next generation genetic sequencing and induced pluripotent stem cell technology are enabling significant acceleration of discovery and understanding. Improved disease understanding begets the emergence and rigorous study of potential treatments, as we are realising for ADPKD [27-30], Alport Syndrome (AS) [31-38], and Tuberous Sclerosis Complex (TSC) [39-42]. Even for more rare forms of GRD such as atypical haemolytic uremic syndrome (aHUS) [39-42] and Fabry’s disease (FD) [43-47], new therapeutic modalities are emerging to challenge historical paradigms of unavoidable decline in renal function, excess burdens of morbidity and mortality, and decreased quality of life.

The spectrum of GRD seen in the adult versus paediatric populations is likely to be different. Autosomal Dominant Polycystic Kidney Disease (ADPKD) is the most striking example, with clinical disease onset and diagnosis largely in adulthood [48] and relatively high disease prevalence at 1/400-1000 population [49-55]. Compared to paediatric populations, ADPKD imparts a significant impact upon GRD prevalence estimates amongst adults.

In order to more precisely profile GRD in an adult CKD population, we have utilised a large and current Australian population based dataset. The CKD.QLD registry is a research platform of the Chronic Kidney Disease in Queensland collaboration (CKD.QLD), formed in 2009 in the third most populous Australian state/territory. It is a collaborative, multidisciplinary research and practice improvement network, which encompasses all public hospital adult nephrology practices across Queensland, which are estimated to provide services to 10,800 CKD patients. This report describes the prevalence of GRD in the first 2,935 consented patients at five sites. These five sites currently provide primary public nephrology services for an estimated 58% of the Queensland resident population, and represent inner metropolitan, outer metropolitan, regional and rural settings. This size and diversity of service locations increases data sensitivity, power and translatability. Understanding of the epidemiology of CKD more broadly and GRD more specifically is hypothesised to be a pre-requisite for effective health service provision, planning and realignment, and for identification of locally unaddressed research priorities.

## Methods

The CKD.QLD registry of all consented patients at five sites as at December 2013 was examined. All patients had clinically diagnosed CKD, were 18 years or older and had attended public nephrology practices in Queensland. Information on gender, age, renal function, CKD stage, and CKD aetiology was available. CKD aetiology was recorded and coded according to the coding protocols of the Australia and New Zealand Dialysis and Transplant registry (ANZDATA). Up to four primary renal diagnoses were listed for each patient and reflected the clinical diagnoses provided by their treating clinicians.

All potentially genetic or inheritable forms of renal disease were extracted from the CKD.QLD registry, and compared with all other CKD patients in the registry. Cases were found by key word searches of the entire registry followed by systematic and exhaustive manual review of all records in the registry. The search included, but was not limited to, the key words ADPKD, autosomal recessive polycystic kidney disease (ARPKD), nephronophthisis, syndromic ciliopathies, Alport Syndrome (AS), thin basement membrane nephropathy (TBMN), medullary cystic kidney disease (type 1, 2 and unspecified; including autosomal dominant tubulointerstitial kidney disease, familial juvenile hyperuricemic nephropathy and UMOD nephropathy), tuberous sclerosis complex (TSC), Fabry Disease (FD), atypical haemolytic uremic syndrome (aHUS), nail-patella syndrome, familial focal segmental glomerulosclerosis (FSGS), Bartter Syndrome, Gitelman Syndrome, and congenital anomaly of the kidney and urinary tract (CAKUT). The inclusion of physician-diagnosed CAKUT (including vesico-ureteric reflux) is open to discussion as described by Fletcher et al. [9] in their study of the prevalence of GRD in the Australian paediatric population. This group of patients has also been included in this study (despite the variable phenotypes), in recognition of the clear description and ongoing discovery of causative genes. Further, their inclusion aids comparison to the local paediatric study, for a more representative and similarly aligned overview of GRD across the lifespan.

Data were collected on the number of patients with each condition, their gender, age (overall and 5 year age groups), estimated glomerular filtration rate (eGFR, ml/min/1.73 m2; CKD-EPI equation), and current CKD stage at consent. These were analysed in comparison to all other CKD.QLD registry patients as well as in subgroups using relative risk (p value and 95% confidence intervals), chi2 (x2) for significance, and T-test or Mann–Whitney Test where applicable. Longitudinal data on renal dysfunction progression were collected and analysed for the subgroup of patients from one site where this was additionally available (ΔeGFR). This single site group constituted 956 patients, representing 32.6% of the total CKD.QLD cohort of 2,935 patients.

For comparison with the incident Australian and New Zealand RRT cohorts, data were extracted from the published ANZDATA registry reports 2001 to 2012 inclusive (http://www.anzdata.org.au), which describes all incident RRT patients from 01.01.2000 to 31.12.2011. Using the paediatric (aged <18 yrs) primary renal diagnosis data from two six year periods (2000–2005 and 2006–2011) and primary renal diagnosis data for all incident patients from each individual year 2000 to 2011, an extrapolation of the adult (aged ≥18 years) GRD and non-GRD cohorts could be made for each of these six year periods. These cohorts were then compared with the GRD and non-GRD CKD.QLD cohorts using relative risk and chi2 (x2) for significance.

Prevalence was calculated as a proportion of the total CKD.QLD population (percentage) and as a proportion of the population 18 years or older (number per million population) served by the 5 sites according to the Queensland Health 2013 Hospital and Health Service boundaries (http://www.health.qld.gov.au) as at the 2011 census (http://www.abs.gov.au).

## Results

### CKD and general population prevalence

A total of 287 patients had a GRD Primary Renal Diagnosis, representing 9.8% of the total cohort of 2,935 adults with CKD. The most common specific GRD diagnoses (Table 1) were ADPKD (4.3%), CAKUT (3.6%), and MCKD (0.5%).

**Table 1:** Specific genetic renal diseases in the adult CKD population (n = 2935)

**Genetic renal disease**	**Number**	**CKD cohort prevalence**	General population prevalence (per million population 18 + yrs)*	**eGFR mean/median (ml/min/m2)**	**Age mean/median (years)**	**Female**
Autosomal dominant polycystic kidney disease	127	4.3%	60.88	50.72/46	53.37/53.53	51.18%
Congenital anomalies of the kidney and urinary tract	106	3.6%	50.81	41.74/38	54.52/56.90	48.11%
Medullary cystic kidney disease	15	0.5%	7.19	45.87/39	48.65/49.27	60%
Thin basement membrane nephropathy	12	0.4%	5.75	82.33/91	51.04/57.01	91.67%
Alport syndrome	5	0.2%	2.40	85.4/91	29.46/25.61	80%
Tuberous sclerosis complex	5	0.2%	2.40	64.4/69	56.34/51.63	100%
Atypical hemolytic uremic syndrome	3	0.2%	2.40	45.67/44	36.66/41.44	100%
Birt-Hogg-Dube syndrome	3	0.1%	1.44	69.33/91	55.79/52.75	100%
Membranoproliferative Glomerulonephritis Type 2 (“Dense deposit disease”)	2	0.1%	0.96	48/48	61.25/61.25	50%
Fabry disease	2	0.1%	0.96	88/88	57.77/57.77	50%
Nephronophthisis	2	0.1%	0.96	40.5/40.5	25.46/25.46	50%
Familial focal segmental Glomerulosclerosis	2	0.1%	0.96	65/65	30.50/30.50	50%
Gitelman syndrome	1	0.1%	0.48	91/91	27.89/27.89	100%
Nail Patella syndrome	1	0.1%	0.48	27/27	30.30/30.30	0%
Renal tubular acidosis	1	0.1%	0.48	20/20	81.28/81.28	0%
**TOTAL**	**287**	**9.8%**	**137.58**	**49.72/45**	**52.6/53.39**	**54.01%**

*Based upon the 2011 Australian bureau of statistics population of 2,086,055 persons aged 18 years or over residing in the areas served by the 5 sites included.

The general population prevalence of CKD derived from this CKD cohort is 1,407 cases per million population 18 years or over. The population prevalence of CKD due to GRD is 138 cases per million population 18 years or over (Table 1).

### CKD population characteristics

There were GRD cases present at all 5 sites with variable distribution (Table 2). One site (Site 1) had the largest number of CKD patients overall, as well as the highest absolute number and proportion of GRD, which was significant compared to the other 4 sites (13.7% vs 7.5%; x^2^ < 0.0005; Relative Risk 1.4, 95% CI 1.2-1.6). Conversely, Site 3 had the second largest number of CKD patients overall but with a significantly lower proportion of GRD (6.4% vs 11.4%; x^2^ < 0.0005; Relative Risk 0.6, 95% CI 0.5-0.8).

**Table 2:** GRD (N = 287) versus non-GRD (N = 2648) CKD population characteristics

**Ckd.Qld Sites**						
	***GRD***		***Non-GRD***		***GRD Vs Non-GRD***	
Site	Number	Proportion of site	Number	Proportion of site	x2	Relative risk (95% Ci)
1	149	13.68%	940	86.32%	<0.0005	1.380 (1.221-1.560)
2	30	7.26%	383	92.74%	0.073	0.723 (0.509-1.027)
3	60	6.37%	882	93.63%	<0.0005	0.628 (0.498-0.791)
4	24	11.53%	184	88.46%	0.396	1.203 (0.801-1.809)
5	24	8.48%	259	91.52%	0.464	0.855 (0.573-1.275)
**Gender**						
	***GRD***		***Non-GRD***		***GRD vs Non-GRD***	
	Number	Proportion of GRD	Number	Proportion of Non-GRD	x2	Relative risk (95% CI)
Male	132	0.46	1440	0.54	0.007	0.738 (0.592-0.921)
Female	155	0.54	1208	0.46	0.007	1.354 (1.086-1.688)
**CKD Stage**						
	***GRD***		***Non-GRD***		***GRD vs Non-GRD***	
CKD stage	Number	Proportion of GRD	Number	Proportion of Non-GRD	x2	Relative risk (95% CI)
1	45	15.68%	134	5.06%	<0.0005	2.714 (2.052-3.591)
2	51	17.77%	277	10.46%	<0.0005	1.718 (1.298-2.273)
3a	48	16.72%	475	17.94%	0.628	0.926 (0.689-1.244)
3b	64	22.30%	887	33.50%	<0.0005	0.599 (0.458-0.782)
4	53	18.47%	685	25.87%	0.006	0.674 (0.506-0.898)
5	23	8.01%	182	6.87%	0.542	1.160 (0.776-1.734)
unknown	3	1.05%	8	0.30%	0.085	2.808 (1.063-7.417)
					T Test/Mann–Whitney Test	
Mean eGFR	49.72		40.35		t (2921) = 7.05, p <0.001	
Median eGFR	45		37		U (2923) = 445087, Z = 5.3, p <0.001	
**Age**						
	***GRD***		***Non-GRD***		***GRD vs Non-GRD***	
Mean age	52.6		69.3		t (2933) = 14.65, p <0.001	
Median age	53.39		66.42		U (2935) = 545319, Z = 12.12, p <0.001	
Age group	Number	Proportion of GRD	Number	Proportion of Non-GRD	x2	Relative risk (95% CI)
15-20	10	3.48%	8	0.30%	<0.0005	5.850 (3.813-8.977)
21-25	20	6.97%	20	0.76%	<0.0005	5.421 (3.896-7.543)
25-30	19	6.62%	40	1.51%	<0.0005	3.456 (2.346-5.091)
31-35	17	5.92%	59	2.23%	0.001	2.369 (1.535-3.656)
36-40	16	5.57%	81	3.06%	0.027	1.727 (1.088-2.741)
41-45	25	8.71%	89	3.36%	<0.0005	2.110 (1.465-3.039)
46-50	24	8.36%	118	4.46%	0.005	1.795 (1.224-2.631)
51-55	33	11.50%	140	5.29%	<0.0005	2.074 (1.493-2.881)
56-60	14	4.88%	233	8.80%	0.024	0.558 (0.331-0.940)
61-65	25	8.71%	296	11.18%	0.232	0.777 (0.524-1.152)
66-70	31	10.80%	373	14.09%	0.126	0.759 (0.531-1.085)
71-75	21	7.32%	441	16.65%	<0.0005	0.423 (0.274-0.652)
76-80	16	5.57%	350	13.22%	<0.0005	0.414 (0.253-0.678)
81-85	12	4.18%	282	10.65%	0.001	0.392 (0.223-0.690)
86-90	2	0.70%	107	4.04%	0.005	0.182 (0.046-0.721)
91-95	2	0.70%	10	0.38%	0.621	1.709 (0.480-6.087)
96-100	0	0%	1	0.04%	1	0.000 (0.000-0.000)

There were significantly more women with GRD as compared to the non-GRD CKD population (54.0% vs 45.6%; x^2^ 0.007; Relative Risk 1.4, 95% CI 1.1-1.7). Both mean and median age of GRD patients was younger than those with CKD not due to GRD (52.6 yrs vs 69.3 yrs, t(2933) = 14.7, p < 0.001; 53.4 yrs vs 66.4 yrs, U(2935) = 54519, Z = 12.1, p < 0.001). Those with GRD were represented in significantly greater proportions in all 5 yr age groups below 56 yrs that were analysed.

**Figure 1:**
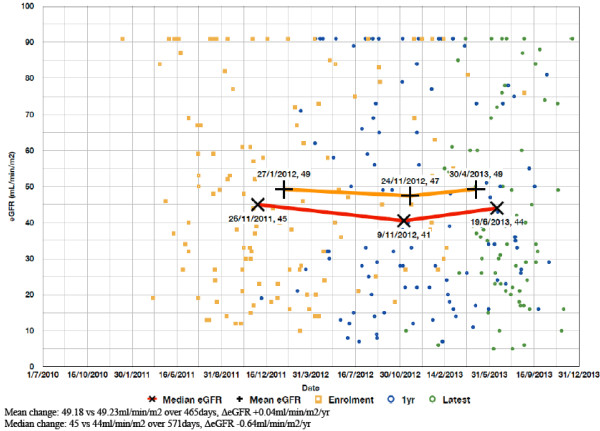
**Change in eGFR over time in Site 1.** GRD patients (n = 120).

**Figure 2:**
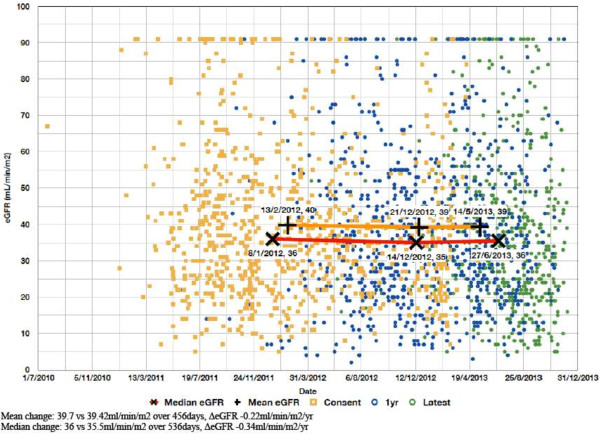
**Change in eGFR over time in Site 1.** Non-GRD patients (n = 834).

Both mean and median eGFR were higher in those with than without GRD (49.7 ml/min/1.73 m2 vs 40.4 ml/min/1.73 m2, t(2921) = 7.1, p < 0.001; 45 ml/min/1.73 m2 vs 37 ml/min/1.73 m2, U(2923) = 445087, Z = 5.3, p < 0.001). Those with GRD were more likely to have CKD stage 1 or 2 (15.7% vs 5.1%, x^2^ < 0.0005, Relative Risk 2.7, 95% CI 2.1-3.6; 17.8% vs 10.5%, x^2^ < 0.0005, Relative Risk 1.7, 95% CI 1.3-2.3) and those without GRD were more likely to have CKD stage 3b or 4 (22.3% vs 33.5%, x^2^ < 0.0005, Relative Risk 0.6, 95% CI 0.5-8; 8.0%% vs 25.9%, x^2^ 0.006, Relative Risk 0.7, 95% CI 0.5-0.9).There was no clear difference in either mean or median ΔeGFR within or between GRD and non-GRD populations for the subset of patients from Site 1 (Figures 1; 2) for whom data on data on renal dysfunction progression were available.

Major non-GRD causes for CKD included renovascular disease including hypertension (24.26%, n = 712), diabetic nephropathy (22.66%, n = 665), uncertain diagnosis (15.06%, n = 442), glomerulonephritis not otherwise specified (3.30%, n = 97), membrano-proliferative glomerulonephritis (2.18%, n = 64), loss of single kidney (2.20%, n = 64), analgesic nephropathy (1.90%, n = 56), focal segmental glomerulonephritis (1.53%, n = 45), bilateral renal artery stenosis (0.99%, n = 29), lupus nephritis (0.95%, n = 28), membranous nephropathy (0.78%, n = 23), lithium nephropathy (0.65%, n = 19), interstitial nephritis (0.51%, n = 15), anti-neutrophil cytoplasmic antibody associated nephropathy (0.37%, n = 11), IgA nephropathy (0.34%, n = 10), and Goodpasture Syndrome (0.2%, n = 6).

### Comparison between CKD and RRT populations

GRD was the Primary Renal Diagnosis in 11.3% and 17.5% of incident Australian and New Zealand Adults commencing RRT between 2006–2011 and 2000–2005 respectively (Table 3). The prevalence of GRD in the CKD.QLD prevalent Australian adult CKD population was significantly lower compared to both cohorts of incident RRT patients (2006–2011: 9.8% vs 11.3%, x^2^ 0.014, Relative Risk 0.9, 95% CI 0.8-1.0; 2000–2005: 9.8% vs 17.5%, x^2^ < 0.0005, Relative Risk 0.6, 95% CI 0.5-0.7). In both time periods there was a significantly higher proportion of paediatric compared to adult cases of GRD incident RRT.

**Table 3:** GRD in published ANZDATA incident RRT

	**01.01.2000 to 31.12.2005**			**01.01.2006 to 31.12.2011**		
**Primary renal diagnosis**	**All incident RRT**	**Paediatric incident RRT**	**Adult incident RRT**	**All incident RRT**	**Paediatric incident RRT**	**Adult incident RRT**
*PKD*	873	7	866	1106	13	1093
*MCGN-II*	27	0	27	28	0	28
*Familial GN (Alport & Secondary FSGS)*	106	12	94	130	4	126
*Cystinosis*	9	7	2	3	2	1
*Oxalosis & Fabry Disease*	12	0	12	9	0	9
*MCKD*	80	17	63	73	8	65
*HUS*	52	6	46	59	12	47
*CAKUT*	637	111	526	674	107	567
*TSC*	3	0	3	1	0	1
*Alagille Syndrome*	0	0	0	1	0	1
*NPS*	1	0	1	1	0	1
*SRNS*	12	0	12	7	0	7
*BHD & VHL*	5	0	5	1	0	1
*Fanconi RTA*	11	0	11	8	0	8
**TOTAL GRD**	1828	160	1668	2101	146	1955
**TOTAL Non-GRD**	9860	357	9503	17584	308	17276
**% of Cohort GRD**	18.52%	44.82%	17.53%	11.94%	47.40%	11.31%
**Adult GRD CKD vs GRD Incident RRT**	9.8% vs 17.53%, x^2^ < 0.0005, Relative risk 0.582, 95% CI 0.520-0.651			9.8% vs 11.31%, x^2^ 0.014, Relative risk 0.869, 95% CI 0.790-956		
**Paediatric vs Adults GRD Incident RRT**	44.81% vs 17.53%, x^2^ < 0.0005, Relative risk 3.574, 95% CI 2.919-4.375			47.4% vs 17.53%, x^2^ < 0.0005, Relative risk 6.645, 95% CI 5.338-8.272		

## Discussion

Here we describe the prevalence of adults with chronic kidney disease due to genetic or inheritable aetiologies within a significant multi-site Australian CKD registry. This confirms this prevalence to be 9.8%, which is not dissimilar to historical estimates [2-4], and provides an evidence-based measurement where one has not been previously clearly described. Furthermore, this finding is clinically similar to, though significantly less than, the 11.3% prevalence of GRD in the most recent cohorts of patients commencing RRT in Australia and New Zealand. Together these suggest that in an Australasian context, approximately 1 in 10 patients with recognised renal disease coming to the attention of renal specialists would be expected to have a genetic aetiology.

Some regional differences may exist, as evidenced by inter-site differences within the CKD cohort studied. These might be explained by differing clinician diagnostic propensities, referral bases, primary care referral models, available clinical services, and clustering of extended affected families by region. The explanation for female preponderance in the GRD population, compared to the male majority in the non-GRD population, is unclear. It would however be expected that many of the female patients with potentially X-linked diseases such as either AS or TBMN would be more likely to have CKD rather than ESKD. This was observed in our study, however these groups accounted for less than 6% of the GRD cohort. A near even gender balance was observed in the 81% of the GRD cohort accounted for by the two largest GRD sub-groups (ADPKD, CAKUT). This suggests that the increased female representation in some small GRD sub-groups may be due to disease specific features of inheritance, rather than other factors, and that this gender bias amongst a minority is responsible for the overall observation of female predominance.

Unsurprisingly those with GRD were significantly younger, in keeping with a genetic aetiology and renal pathobiology progressing throughout the lifespan and commencing early in life. An additional cause may be younger age of diagnosis and commencement of renal follow-up due to more proactive screening triggered by knowledge of affected family members. This could also explain the better preserved renal function observed in those with GRD. The absence of clearly more rapid renal dysfunction progression in the small and short-term single site cohort of patients suggests that patients with GRD are presenting to nephrologists earlier than those without GRD, either as a result of known family history, symptoms not exhibited best by renal dysfunction, or even extra-renal phenotypes. This group would also be less likely to have a pre-existing background of cardiovascular disease or metabolic dysfunction that may be associated with renal function decline.

The cause for the observed decrease in adult GRD incident RRT from 2000–2005 (17.5%) to 2006–2011 (11.3%) is not clear. During this time period there was minimal change in the paediatric GRD incident RRT cohort prevalence (Table 3). One explanation is a dilutional effect exerted by non-GRD causes for renal disease amongst adults due to increasing prevalence and/or more rapid renal dysfunction progression. A candidate for this is diabetic nephropathy, the proportion of which increased within the incident RRT cohort in a sustained and inexorable fashion from 24.8% in 2000 to 36.5% in 2011. Even though absolute numbers of incident RRT due to GRD in adults increased from 1,668 in 2000–2005 to 1,955 in 2006–2011 this was modest by comparison to vastly increasing overall numbers of overall adult RRT patients (9,514 in 2000–2005 to 17,284 in 2006–2011). No historical data on primary renal diagnosis are available for this CKD cohort during that 12 year timeframe of considerable change within the relevant RRT population. Ongoing CKD cohort surveillance and future analyses will be important to enable hypothesis generation in the future.

Some GRD’s were notably absent in this cohort, including Dent Disease, Renal Cysts and Diabetes (RCAD) Syndrome, Cystinuria, Bartter Syndrome, Familial Hypomagnesaemic Hypercalciuria, and Gordon Syndrome. Whilst the causes for this are not clear, some may be absent due to misdiagnosis (RCAD vs ADPKD; Bartter Syndrome vs Gitelman Syndrome) or being predominantly cared for in non-renal specialty clinics (Gordon Syndrome in Hypertension Clinic; Cystinuria in Urology). There is also some chance that population prevalence may be very low. The diagnoses in this registry are clinician derived and it is not clear as to the proportion of reported GRD cases that have been confirmed with a molecular genetic diagnosis. Despite this, the major two GRD’s (ADPKD, CAKUT) are predominantly diagnosed clinically, even in the absence of a confirmatory molecular genetic diagnosis.

The population prevalence of GRD within the adult CKD population that we present as disease prevalence per million aged ≥18 years is likely to be an underestimation in the general community. There are three main causes for this. The first is under-ascertainment, as not all cases of CKD and/or GRD seen in public hospital nephrology practices are being enrolled in CKD.QLD. The second is that some cases of CKD and/or GRD are seen in private nephrology practice and thus not enrolled in the CKD.QLD registry. Lastly, there are likely cases of CKD and GRD resident in the service areas of these 5 sites that remain miscoded, undiagnosed, unknown, or unreferred to any nephrology services. Despite these limitations, the percentage prevalence is however likely to be representative of the broader CKD population. As the CKD.QLD registry grows and matures, and other Australian CKD registries emerge, a larger study would be warranted to confirm and correlate these findings.

## Conclusions

The prevalence of GRD within this CKD population is similar to, although at the lower range, of prior general estimations. These patients are younger with higher levels of renal function and are more likely to be female. Their renal dysfunction was not observed to deteriorate faster than other CKD patients. A significant absolute number and proportion of those starting RRT continue to have GRD. This information is important for multidisciplinary patient counselling and management. The finding of a significant and identifiable minority of patients with CKD having GRD confirms this group as a priority target for optimised future service planning within the emerging paradigm of personalised medicine and healthcare. This also allows the early identification and management of at risk family members, prior to them developing significant renal dysfunction. Future surveillance of this and other CKD cohorts is required to correlate and corroborate these findings as well as to observe future changes.

## Competing interests

Dr Andrew Mallett is a current recipient of a RBWH Foundation Research Postgraduate Scholarship and past recipient of a Churchill Fellowship. He has received a travel grant from Amgen.

The results presented in this paper have not been published previously in whole or part, except in abstract format.

## Authors’ contributions

AM, ZW, AS and WH were involved in design, acquisition and analysis of data, drafted the manuscript and revised critically the manuscript. HH and CP were involved in design of the study and revised the manuscript. All authors have read and approved the final manuscript.
